# Insecticidal and Synergistic Potential of Three Monoterpenoids against the Yellow Fever Mosquito, *Aedes aegypti* (Diptera: Culicidae), and the House Fly, *Musca domestica* (Diptera: Muscidae)

**DOI:** 10.3390/molecules28073250

**Published:** 2023-04-05

**Authors:** Oshneil S. Baker, Edmund J. Norris, Edwin R. Burgess

**Affiliations:** 1 Department of Entomology and Nematology, University of Florida, Gainesville, FL 32611, USA; 2 United States Department of Agriculture, Agricultural Research Service, Center for Medical, Agricultural, and Veterinary Entomology, Gainesville, FL 32608, USA

**Keywords:** *Aedes aegypti*, *Musca domestica*, house fly, toxicology, natural products, insecticide synergists

## Abstract

As resistance to the limited number of insecticides available for medical and veterinary pests becomes more widespread, there is an urgent need for new insecticides and synergists on the market. To address this need, we conducted a study to assess the toxicity of three monoterpenoids—carvone, menthone, and fenchone—in comparison to permethrin and methomyl against adults of two common pests: the yellow fever mosquito (*Aedes aegypti*) and the house fly (*Musca domestica*). We also examined the potential for these monoterpenoids to enhance the effectiveness of permethrin and methomyl when used together. Finally, we evaluated the ability of each monoterpenoid to inhibit acetylcholinesterase, comparing them to methomyl. While all three monoterpenoids performed relatively poorly as topical insecticides (LD_50_ > 4000 ng/mg on *M. domestica*; >6000 ng/mg on *Ae. aegypti*), they synergized both permethrin and methomyl as well as or better than piperonyl butoxide (PBO). Carvone and menthone yielded synergistic co-toxicity factors (23 and 29, respectively), which were each higher than PBO at 24 h. Currently, the mechanism of action is unknown. During preliminary testing, symptoms of acetylcholinesterase inhibition were identified, prompting further testing. Acetylcholinesterase inhibition did not appear to explain the toxic or synergistic effects of the three monoterpenoids, with IC_50_ values greater than 1 mM for all, compared to the 2.5 and 1.7 µM for methomyl on *Aedes aegypti* and *Musca domestica*, respectively. This study provides valuable monoterpenoid toxicity and synergism data on two pestiferous insects and highlights the potential for these chemistries in future pest control formulations.

## 1. Introduction

Resistance to the limited number of insecticides registered for use against medical and veterinary arthropod pests threatens public health and food safety worldwide. Pyrethroids, organophosphates, carbamates, neonicotinoids, and spinosyns are some of the most commonly used chemical classes against pests, such as mosquitoes, face flies, stable flies, and house flies, all with documented combinations of target-site resistance [1,2,3,4], enhanced metabolic detoxification [5,6,7], reduced cuticular penetration [8,9], and behavioral resistance [10,11,12]. Synergists such as piperonyl butoxide (PBO) can restore the efficacy of some of these chemical classes when metabolic detoxification is a major mechanism [13]. No new chemical classes or synergists have come to market recently for medical and veterinary pests, highlighting a need for exploration of both types of chemicals.

Monoterpenoids are plant-produced secondary metabolites characterized by their volatility and fragrant odor. Carvone, a monoterpenoid abundantly found in caraway, spearmint, and dill seeds [14], has shown insecticidal efficacy under lab conditions against stored grain pests such as *Sitophilus oryzae* (Coleoptera: Curculionidae), *Rhyzopertha dominica* (Coleoptera: Bostrichidae), and *Tribolium castaneum* (Coleoptera: Tenebrionidae) as both a contact and fumigant toxicant [15]. Fenchone, a monoterpenoid extracted from absinthe and fennel, was found to be a contact toxicant for three tested stored grain pests [16]. Interestingly, monoterpenoids have rarely been screened as synergists for medical and veterinary pests. The volatility and contact toxicity of monoterpenoids make them appealing for medical and veterinary control because most applications involve space or residual sprays or ultra-low volume (ULV) fogging. Two medical and veterinary pests that are frequently controlled with contact toxicants through sprays or fogging are the yellow fever mosquito, *Aedes aegypti* (Diptera: Culicidae), and the house fly, *Musca domestica* (Diptera: Muscidae).

The yellow fever mosquito, *Ae. aegypti*, is a synanthropic pest known to preferentially feed on humans [17] and will take multiple blood meals per gonotrophic cycle [18], enhancing their potential to vector pathogens. Notable examples of pathogens spread by *Ae. aegypti* include yellow fever, dengue, chikungunya, and Zika viruses, which are among the most historically impactful arthropod-borne human pathogens [19]. Widespread resistance to insecticides has been documented in *Ae. aegypti* [20], with all tested Florida *Ae. aegypti* strains being resistant to permethrin compared to a susceptible laboratory colony. Resistance ratios ranged from 6-fold to 61-fold in field strains in comparison to the lab strain [21].

The house fly, *M. domestica*, is a synanthropic pest known to mechanically transmit more than 100 pathogens that cause diseases in both humans and animals [22]. *Musca domestica* can transmit bacteria that cause mastitis in lactating dairy cows and *Salmonella* spp. (Enterobacteriales: Enterobacteriaceae) within both swine and poultry facilities [23,24]. Between bacterial infection, irritation, and food spoilage, *M. domestica* is responsible for losses exceeding $30 million in the poultry industry, $135 million in the dairy industry, and $35 million in the swine industry [25]. Within urban settings, *M. domestica* can transmit bacteria found on farms and may cause a severe nuisance from up to 3.2 km away from a typical layer facility [26]. A US survey of pyrethroid resistance in *M. domestica* found highly resistant flies nearly everywhere they were sampled, including populations that overexpress cytochrome P450 as a metabolic mechanism of resistance [27].

The objective of this study was to investigate the contact toxicity and synergistic effects of three monoterpenoids—menthone, fenchone, and carvone—on both *Ae. aegypti* and *M. domestica* adults. Initial screening efforts presented a symptomology consistent with acetylcholinesterase inhibition, and we also explored the acetylcholinesterase inhibitory potential of these monoterpenoids compared to methomyl, an insecticide found in baits against *M. domestica* and belonging to the carbamate class of acetylcholinesterase inhibitors.

## 2. Results

### 2.1. Topical Dose Response

Overall, the monoterpenoids were less toxic to both *Ae. aegypti* and *M. domestica* compared to methomyl and permethrin (Table 1). Among the monoterpenoids, carvone and menthone were statistically equivalent and had greater toxicity on *Ae. aegypti* at LD_10_, LD_50_, and LD_90_ compared to fenchone. Menthone was about 1.5 times as toxic as fenchone at LD_10_ and LD_50_ and about 4.2 times as toxic at LD_90_. Carvone was about 1.9 times as toxic at LD_10_, 1.5 times as toxic at LD_50_, and about 3.0 times as toxic at LD_90_. Fenchone was also the least toxic of the three monoterpenoids in *M. domestica*, and carvone was the most toxic.

In *M. domestica,* at the LD_10_, carvone was 1.6 and 2.7 times as toxic as menthone and fenchone, respectively. Carvone was 1.6 and 3.1 times as toxic as menthone and fenchone, respectively, at the LD_50_. Carvone was 1.6 and 3.6 times as toxic as menthone and fenchone, respectively, at the LD_90_. After the mg of body weight was corrected, there were some notable differences among the LD values between species. Permethrin was significantly more toxic to *Ae. aegypti* than to *M. domestica* at the LD_10_ and LD_50_ but not at the LD_90_. However, carvone appeared to be more toxic to *M. domestica* at the LD_50_ and LD_90_ compared to *Ae. aegypti*. Similarly, fenchone was more toxic to *M. domestica* at the LD_90_.

### 2.2. Co-Toxicity Assays

The synergistic capabilities of our tested monoterpenoids expressed great potential. The effect of the synergistic mixtures of PBO and all monoterpenoids was more pronounced at the 24-h mortality compared to permethrin-only doses in *M. domestica*. For *Ae. aegypti*, the effects of synergistic mixtures of PBO and carvone were less pronounced, with PBO being the least pronounced (at the 2 µg/insect dose). Notably, menthone and fenchone synergist mixtures at 24-h mortality expressed effects that were greatly more pronounced than those of permethrin alone.

This difference was even more pronounced at 24-h mortality, where fenchone was about 6.4 times as strong of a synergist to permethrin as PBO was in *Ae. aegypti* (Table 2). Both menthone and carvone were also superior 24-h mortality synergists compared to PBO in *Ae. aegypti*. At 2 μg of synergist, the effect was greater than when 10 μg was applied. The synergism of 24-h mortality with PBO at 10 μg could not be calculated because of high mortality produced by the synergist alone, which has been seen before [28]. For *M. domestica*, the synergism of knockdown was only produced by fenchone, but all other compounds were additives to permethrin knockdown. For 24-h mortality, all tested compounds were synergistic, with carvone and fenchone acting as slightly superior synergists compared to PBO, and menthone acting as a slightly inferior synergist.

The synergism of monoterpenoids and PBO with methomyl was only tested in *Ae. aegypti* (Table 3) because sufficient methomyl toxicity was not observed even at the highest doses applied to *M. domestica* (LD_50_ > 100 ng). In contrast to permethrin, most of the compounds tested were antagonistic or provided no effect on knockdown at either 2 μg or 10 μg. The exception was carvone at 2 μg, which was slightly synergistic (co-toxicity factor = 23.5). At 2 μg of synergist, carvone became synergistic with methomyl at 24- and 48-h mortality. Menthone was synergistic at 24-h mortality but not at 48-h mortality, where it was additive. Fenchone was considerably more antagonistic at 48 h compared to 24 h and 1 h (i.e., knockdown). At 10 μg, all compounds tested were synergistic at 24-h mortality but only additive at 48-h mortality.

### 2.3. In Vitro Inhibition of Acetylcholinesterase (AChE) Activity

None of the monoterpenoids produced the requisite > 50% inhibition to enable the calculation of an IC_50_ value and confidence intervals within the range tested. When corrected for total protein (mg/mL), methomyl produced an IC_50_ (95% CI) of 1.7 (0.7–2.8) µM in the *Ae. aegypti* preparation and 2.5 (2.3–2.7) µM in the *M. domestica* preparation. At the top concentration of 1 mM, each monoterpenoid produced no measurable inhibition in the *Ae. aegypti* preparation, while in the *M. domestica* preparation there was a small inhibitory effect, with carvone showing the greatest inhibitory effect of 11.1 ± 2.9%. Menthone produced 3.7 ± 1.2% inhibition at this concentration, and fenchone produced 1.9 ± 0.3% inhibition at 1 mM. At 100 µM, methomyl produced 99.4 ± 0.1% inhibition.

## 3. Discussion

With a dearth of chemicals available for medical and veterinary pests, the present study indicates that the monoterpenoids menthone, fenchone, and carvone may offer two potentially useful functions against these types of pests. Although the monoterpenoids tested did not perform as well as both permethrin for *Ae. aegypti* and *M. domestica* and methomyl for *Ae. aegypti*, the laboratory strains tested were insecticide susceptible. We noted that carvone generally performed best as a topical toxicant against both species, although menthone was just as good against *Ae. aegypti*. The monoterpenoids were about 10,000-fold less toxic compared to permethrin in both species and about 1000-fold less toxic compared to methomyl in *Ae. aegypti*. With some wild strains of *M. domestica* reaching resistance ratios greater than 5000-fold against permethrin [30], monoterpenoids may have value as an insecticide provided that there is no cross-resistance and they do not have unfavorable toxicological profiles for non-targets, including humans and livestock.

Great care should be taken when referring to plant-derived or other natural compounds as “safe” or “environmentally friendly.” The three monoterpenoids tested have a favorable toxicological profile both in oral and dermal animal testing compared to permethrin and methomyl (Table 4). While monoterpenoids are favorable, they do not lack toxicity and may have possible negative environmental effects. Additional studies may identify sufficiently negative effects if monoterpenoids are used in large quantities.

In terms of oral toxicity, all three monoterpenoids are much less toxic to rats compared to methomyl and slightly less toxic compared to permethrin. Other monoterpenoids similarly have favorable LD_50_ values, such as carvacrol and pulegone, which are within the range of 2000–3000 mg/kg [38]. Moreover, monoterpenoid’s natural volatility increases the rate at which they naturally degrade in the environment. Under simulated outdoor conditions, carvone’s half-life was between 1.8 and 3.2 days, depending on soil type, when it was applied at 5 mg per kg of soil [39]. In acidic conditions, this could increase to as much as 4.5 d. Under a mercury lamp, the half-life was between 0.96–1.16 d, while under a xenon lamp, the half-life was between 3.61 and 4.13 days. Comparatively, the aerobic soil half-life of permethrin is 11.6–113 d [40], while methomyl is approximately 14 d [41]. Low mammalian toxicity combined with fast environmental degradation, at least for carvone, enhances the flexibility of monoterpenoids as potential insecticides. Their volatility makes them potentially good fumigants [15,16], with monoterpenoids currently on the market in this capacity including limonene, linalool, thyme oil, and eugenol. Monoterpenoids are unlikely to be candidates for bait formulation, as they have been found to be both repellants and antifeedants [42].

With how dramatic insecticide resistance has become, the potential of monoterpenoids as synergists may serve to increase the lifespan of current insecticides such as permethrin and methomyl. If monoterpenoids work similarly to other synergists described, such as PBO and MGK-264, this may occur by reducing the effective dose required to cause mortality by inhibiting cytochrome P450s [13]. In turn, lower effective doses of marketed insecticides will remain potent for a longer period of time. Regulatory boards such as the EPA have recommended various stewardship methods to increase the life of our most effective insecticides [43]. These include the rotation of insecticides and using insecticides with multiple modes of action. Synergists will likely be a key addition to the stewardship of our current insecticides. Monoterpenoids such as the ones tested may add to the limited pool of synergists currently available on the market.

Piperonyl butoxide (PBO) and MGK-264 are the most common synergists on the market. These registered synergists typically work by blocking the activity of metabolic enzymes that detoxify insecticides [44]. Synergists have been commercially successful for over 50 years and are commonly used to aid in both managing and possibly reversing resistance [45,46]. However, MGK-264 is highly controlled due to its characterized toxicity, which leaves PBO as the most common synergist used. It should be noted that even PBO’s safety has been questioned [28,47]. Monoterpenoids, however, show remarkable safety as many are used in products such as candles and food.

Menthone, fenchone, and carvone were surprisingly good synergists. When tested in *Ae. aegypti*, fenchone + permethrin were 6.4 times more potent than PBO + permethrin, despite possessing the lowest toxicity among all monoterpenoids. Menthone and carvone followed at 5.3 and 3.3 times, respectively. All monoterpenoids tested exhibited significant increases in mortality over PBO when combined with permethrin and methomyl. This seems to be dependent on species, however, as differences in synergistic capability were significantly decreased in *M. domestica*. When tested with permethrin, carvone was only 1.2 times as potent as PBO in comparison to *Ae. aegypti*. Fenchone and menthone followed with 1.1 and 0.9 times, respectively. Within *M. domestica*, menthone was less synergistic than PBO yet highly synergistic in *Ae. aegypti*. This may hint at the monoterpenoids being better suited as synergists in ULV or similar mosquito sprays. Despite this, PBO is a highly effective synergist in *M. domestica* control products. That the monoterpenoids showed similar synergism to PBO against *M. domestica* is not an indictment against any of the monoterpenoid’s ability to act as synergists.

When synergized with methomyl and tested on *Ae. aegypti*, menthone and carvone were 2.0 and 1.7 times as effective as PBO, respectively, while fenchone exhibited a negative co-toxicity factor. Our data suggest that monoterpenoid synergism may be highly dependent on both the target organism and the active ingredient. However, generally speaking, both menthone and carvone served as more potent synergists compared to PBO.

In preliminary testing, it was observed that dosed flies and mosquitoes expressed some of the common symptoms of an acetylcholinesterase inhibitor, much like methomyl. These include characteristic behaviors such as hyperactivity, uncoordinated movement, and convulsions [48]. While monoterpenoids can be converted to *n*-methyl carbamates in the presence of methyl isocyanate and a catalytic amount of triethylamine [49], our evidence suggests they do not share a mode of action with carbamates. Therefore, acetylcholinesterase inhibition shows limited toxicological relevance, at least in terms of describing a primary mode of action.

Monoterpenoids offer manufacturers a promising source of new potential insecticides and insecticide synergists. Future research should focus on expanding information on the synergistic capabilities of monoterpenoids, including with other active ingredients. In the advent of pesticide resistance of global magnitude, synergistic monoterpenoids may serve as a great equalizer of pest resistance.

## 4. Materials and Methods

### 4.1. Insects and Chemicals

The CAR21 susceptible strain of *M. domestica* used in this study was obtained from the USDA-ARS Center for Medical, Agricultural, and Veterinary Entomology (CMAVE). All the flies were 3–5-day-old adults during testing and were allowed to feed on sucrose and water ad libitum. The Orlando strain of *Ae. aegypti* was also obtained from USDA-ARS-CMAVE and reared under standard laboratory rearing protocols. l-menthone (97%), l-fenchone (>98%), l-carvone (98%) (hereafter referred to as “menthone”, “fenchone”, and “carvone”), and all chemicals for the acetylcholinesterase inhibition assay were obtained from Fisher Scientific (Waltham, MA, USA). Doses were formulated utilizing the densities of each monoterpenoid at 25 °C (i.e., room temp); l-carvone 0.96 g/mL, l-menthone 0.895 g/mL, and l-fenchone 0.948 g/mL. Permethrin (99.7% pure, 77.8% trans, and 21.9% cis) and methomyl (99.5% purity) were from Chem Service (West Chester, PA, USA).

### 4.2. Topical Dose Responses

For the *Ae. aegypti*, topical applications of solutions containing monoterpenoids or insecticides were performed using similar methods to those outlined in Norris et al. [50]. In short, adult female mosquitoes were aspirated using an InsectaVac aspirator (BioQuip, Claremont, CA, USA) and then subsequently anesthetized on ice prior to the application of insecticidal solution. Mosquitoes were held on a cold glass petri dish to prevent reanimation, and a Whatman No. 2 filter paper was used to prevent excess condensation on the Petri dish. Only mosquitoes aged between 3 and 7 days post-eclosion were used for this study. Solutions of monoterpenoids or insecticides were made in ethanol, and 0.2 µL of differing concentrations were applied to the pronotum of mosquitoes using a Hamilton repeating applicator and a 10 µL Hamilton syringe (Hamilton, Reno, NV, USA). At least 10 mosquitoes were utilized for each concentration tested, representing a single replicate, and at least 3 distinct rearing cohorts (reared from separate egg batches) were used for each concentration screened. The treated mosquitoes were then transferred to a 16-ounce deli cup with tulle fabric placed over the top to prevent escape. Mosquitoes were then transferred to an incubator and maintained at a constant temperature of 28 ± 2 °C with a light cycle of 12:12 h light: dark. The humidity was maintained at a relatively constant 75 ± 10% RH using a water pan placed at the bottom of the incubator. Only non-blood-fed mosquitoes were used in the assays. A minimum of four concentrations were used for each dose-response curve for each treatment. Treated mosquitoes were held for 48 h post-application, with toxicity observed at 1 h (knockdown), 24 h (mortality), and 48 h (mortality) after applying insecticide. Knockdown was defined as the inability to fly or maintain normal standing posture, and mortality was defined by ataxia after the rapping of the assay container.

For *M. domestica*, 20 female flies were utilized per dose, with at least three separate rearing cohorts used, the same as for *Ae. aegypti*. Flies were first vacuumed from age-controlled cages and anesthetized with CO_2_. Flies were sorted by sex under this anesthesia and placed in glass petri dishes (100 × 20 mm). All flies were allowed to fully recover from anesthesia prior to testing. To dose, the glass petri dishes containing flies were anesthetized with ice. The petri dishes full of anesthetized flies were then transferred to a small Pyrex casserole pan filled with ice. A 0.5 µL droplet of insecticide treatment in acetone was deposited on the dorsal thoracic notum of each fly. Dosed flies were then transferred to 250-mL flint jars and covered with fiberglass screen material. A cotton ball saturated with a 20% sucrose solution was placed on the mesh. Flies were assessed for knockdown at 1 h and mortality at 24 h. All dosed flies were held at 25 ± 2 °C and 60 ± 5% RH. Knockdown was defined as described previously for *Ae. aegypti*. Mortality was scored when flies could not regain a standing position when lying on their backs or sides or were nonresponsive to gentle shaking of the test jars.

### 4.3. Co-Toxicity Assays

For *Ae. aegypti*, synergism assays were performed similar to the topical applications described previously, with the following modifications: 2 doses (2 µg/mosquito and 10 µg/mosquito) of each monoterpenoid were applied as synergists, similar to previous studies exploring the synergistic potential of natural products in combination with an intermediate dose-level of insecticide alone [51]. The concentrations of permethrin (0.6 ng/mosquito) and methomyl (6 ng/mosquito) used were chosen as they produced an average mortality among replicates between 10 and 75% at 24 h, with a 24-h mortality of 36 ± 6% and 57.5 ± 25.3%, respectively. For PBO, only the 2 µg/mosquito dose level was used to assess synergism, as the mortality of PBO alone at 10 µg/mosquito was too high (72.5 ± 8.5%) to adequately assess the synergistic effect using co-toxicity factor analysis.

$$\text{co-toxity}\;\mathrm{factor}\;=\;\frac{observed\%mortality-expected\%mortality}{expected\%mortality}\times 100$$

Knockdown was observed at 1 h, and mortality was observed at both 24 and 48 h post-application. Mosquitoes were transferred to deli cups and kept at a controlled temperature and humidity (the same conditions as described for the topical application of insecticides and monoterpenoids alone). Again, a minimum of 3 separate rearing cohorts were used among replicates of each dose combination.

For *M. domestica*, near-sublethal doses of PBO 19.44 nL/µL (10.2 µg), carvone 145.8 nL/µL (70 µg), fenchone 200.4 nL/µL (190 µg), and menthone 178.8 nL/µL (80 µg) were utilized as synergists. Each dose was formulated in a total volume of 1.5 mL of acetone. A dose of permethrin that produced approximately 50% mortality (18 ng) was used as the treatment. Female flies were sorted into groups of 20 per synergism assay and dosed as previously mentioned (i.e., the same way as in the topical toxicity assays). Flies were assessed for mortality at 24 h.

### 4.4. In Vitro Inhibition of Acetylcholinesterase (AChE) Activity

Acetylcholinesterase inhibition was conducted on homogenates of whole-body *Ae. aegypti* and *M. domestica* heads using Ellman’s method [52]. For *Ae. aegypti*, 10 whole female adults were placed in 2 mL microcentrifuge screw cap tubes with 3–5.2 mm zircon/silica beads and homogenized in 500 μL of sodium phosphate buffer (100 mM, pH 7.8) using a Precellys Evolution bead beater (Bertin Corp., Rockville, MD, USA) set to two 15-s pulses of 5600 rpm. Afterward, 500 μL of Triton X-100 buffer (100 mM sodium phosphate, pH 7.8, 0.6% Triton X-100) was added to each tube (final Triton X-100 concentration: 0.3%), inverted several times to mix up the sample, and then centrifuged at 10,000× *g* for 4 min at 4 °C. The supernatant was used as the enzyme source. The same process was carried out with *M. domestica*, except three heads were processed in a 1000:1000 μL sodium phosphate buffer and Triton X-100 buffer in the same manner as just described.

Concentration responses were conducted in the wells of a 96-well, clear, flat-bottomed plate. Inhibitor solutions of methomyl or monoterpenoids were first dissolved in DMSO (dimethyl sulfoxide) and then diluted in sodium phosphate buffer (100 mM, pH 7.8, DMSO concentration of 1%). A volume of 10 μL of the inhibitor solution was added to each well, along with 90 μL of sodium phosphate buffer, and finally 10 μL of homogenate. This was incubated on a plate shaker at 400 rpm for 10 min at room temperature. The final concentrations for methomyl were 100 μM, 10 μM, 1 μM, 100 nM, 10 nM, 1 nM, and 0 nM. For the monoterpenoids, the same range was used except the top concentration was 1 mM and the bottom non-zero concentration was 10 nM. The final concentration of DMSO in all wells was 0.09%.

After the 10-min incubation, 100 μL of Ellman’s reagent prepared in sodium phosphate buffer and each corresponding inhibitor concentration were added to the wells using a multichannel pipette. This ensured that the molarity of the inhibitor and the DMSO concentration in all wells did not change with the addition of Ellman’s reagent. The final substrate concentrations were 0.4 mM for ATCh (acetylthiocholine iodide) and 0.3 mM for DTNB (5,5′-dithio-bis(2-nitrobenzoic acid). The absorbance of each well was then read every 2 min for a total of 20 min at 405 nm on a BioTek Epoch 2 plate reader (BioTek, Santa Clara, CA, USA). The change in absorbance per minute was calculated in each well and subtracted from wells with no inhibitor (0 nM) to determine inhibition of the reaction rate.

### 4.5. Statistical Analyses

Dose responses were modeled via probit analysis, and their resulting estimates were obtained [53]. A PROC PROBIT analysis was utilized in SAS 9.4 (SAS Institute, Inc., Carey, NC, USA) to calculate LD_10_, LD_50_, and LD_90_ estimates and their corresponding 95% CIs and slopes. A control correction option (OPTC command) was used to account for responses to the vehicle control treatments. Co-toxicity values were calculated by the method of [29]. A co-toxicity factor of >+20 signifies potentiation, <−20 antagonism, and −20 to +20 additive. In vitro AChE inhibition assays were assessed with a four-parameter log-logistic model, and IC_50_ values and 95% confidence intervals were generated with the “drc” package [54] in R version 4.2.0 (R Core Team 2022).

## 5. Conclusions

In this study, we identified topical insecticidal potential of three monoterpenoids, l-menthone, l-fenchone, and l-carvone, including their capabilities to synergize established insecticides used against the house fly, *Musca domestica*, and the yellow fever mosquito, *Aedes aegypti*. While the three monoterpenoids tested were inferior insecticides compared to permethrin and methomyl, in terms of low mammalian toxicity and favorable environmental fates, monoterpenoids may serve in some capacity as standalone insecticides. However, l-menthone, l-fenchone, and l-carvone showed promise as synergists depending on which insecticide they are paired with and at what concentrations they are applied. Future work should focus on characterizing the mechanism of action and synergy when monoterpenoids are used as insecticides or synergists, respectively.

## Figures and Tables

**Table 1:** Twenty-four-hour lethal doses of topically applied monoterpenoids, methomyl, and permethrin in adult females with susceptible strains of *Aedes aegypti* and *Musca domestica*.

	*n*	LD_10_ (95% CI) ^1^	LD_50_ (95% CI) ^1^	LD_90_ (95% CI) ^1^	Slope (SE)
*Ae. aegypti*					
Carvone	220	3900 (1900–5200) b	7300 (6700–7900) c	14,200 (11,000–26,600) c	8.9 (1.6)
Menthone	220	5000 (1000–6300) b	7100 (4100–8300) c	10,000 (8500–13,100) c	8.6 (3.3)
Fenchone	220	7600 (5000–9500) b	11,300 (10,200–53,400) d	42,000 (28,800–94,900) d	11.2 (5.1)
Methomyl	280	0.97 (0.27–1.6) a	2.7 (1.6–4.3) b	7.55 (4.7–22.5) b	2.9 (0.7)
Permethrin	350	0.23 (0.12–0.33) a	0.58 (0.45–0.71) a	1.41 (1.08–2.24) a	3.5 (1.4)
*M. domestica*					
Carvone	480	3300 (3100–3400) b	4300 (4200–4400) b	5600 (5300–5900) b	11.3 (0.9)
Menthone	1280	5300 (5100–5400) c	6800 (6700–7000) c	8800 (8400–9400) c	7.2 (0.5)
Fenchone	600	8800 (8100–9400) d	13,200 (12,600–13,800) d	19,900 (18,600–21,600) d	11.4 (0.8)
Methomyl	-	-	-	-	-
Permethrin	580	0.46 (0.37–0.56) a	0.84 (0.79–0.93) a	1.5 (1.4–1.7) a	5.2 (0.6)

^1^ Units in ng/mg body weight. Different lowercase letters within each lethal dose column indicate statistical significance based on the non-overlap of 95% CIs. *n*—number; LD—lethal dose to kill the subscript denoted percentage of the population; CI—confidence interval; SE—standard error. The mean ± SEM body weight for *Ae. aegypti* was 3.13 ± 0.23 mg/mosquito. The mean ± SEM body weight for *M. domestica* was 21.6 ± 0.36 mg/fly.

**Table 2:** Diagnostic doses and co-toxicity of l-carvone, l-menthone, and l-fenchone with permethrin against *Aedes aegypti* and *Musca domestica*.

	1-h% Mean Knockdown ± SEM				24-h% Mean Mortality ± SEM			
	Permethrin Alone	Synergist Alone	Mixture	Co-Toxicity Factor ^a^	Permethrin Alone	Synergist Alone	Mixture	Co-Toxicity Factor ^a^
*Ae. Aegypti* *								
Control (ethanol)	NA	0 ± 0	NA	NA	NA	0.3 ± 0.1	NA	NA
PBO	87.5 ± 6.3	7.5 ± 2.5	52.5 ± 7.5	−44.7	27.5 ± 4.8	12.5 ± 6.3	50 ± 5.77	25
Carvone	76 ± 9.2	5 ± 5	100 ± 0	23.5	36 ± 6	5 ± 2.9	75 ± 5	83
Menthone	76 ± 9.2	7.5 ± 4.8	100 ± 0	19.8	36 ± 6	5 ± 2.9	95 ± 5	132
Fenchone	76 ± 9.2	0 ± 0	100 ± 0	32.0	36 ± 6	2.5 ± 2.5	100 ± 0	160
*Ae. Aegypti* **								
Control (ethanol)	NA	0 ± 0	NA	NA	NA	0.3 ± 0.1	NA	NA
PBO	76 ± 9.2	22.5 ± 4.8	60 ± 14.7	−39	NA	72.5 ± 8.5	NA	NA
Carvone	76 ± 9.2	20 ± 5.8	100 ± 0	4	36 ± 6	7.5 ± 2.5	90 ± 10	76
Menthone	76 ± 9.2	12.5 ± 4.8	100 ± 0	13	36 ± 6	15 ± 5	85 ± 5	83
Fenchone	76 ± 9.2	10 ± 4.1	100 ± 0	16	36 ± 6	2.5 ± 2.5	85 ± 15	121
*M. domestica* ***								
Control (acetone)	NA	0 ± 0	NA	NA	NA	0.6 ± 0.6	NA	NA
PBO	80 ± 7.3	2 ± 2	96 ± 4	16.2	58 ± 10.1	6 ± 2.9	100 ± 0	55.9
Carvone	80 ± 7.3	16 ± 11.8	100 ± 0	3.2	58 ± 10.1	0 ± 0	96 ± 3.8	65.6
Menthone	80 ± 7.3	4 ± 2.4	95 ± 2.9	12.6	58 ± 10.1	0 ± 0	86 ± 6.6	48.4
Fenchone	80 ± 7.3	0 ± 0	100 ± 0	24.0	58 ± 10.1	0 ± 0	95 ± 2.9	63.4

* Dosed with 2 μg synergist per *Ae. aegypti*. ** Dosed with 10 μg synergist per *Ae. aegypti*. *** *M. domestica* dosed with 10.2 μg PBO, 70 μg carvone, 190 μg menthone, and 80 μg fenchone, which were determined to deliver near-sublethal mortality at 24 h. ^a^ A co-toxicity factor of >+20 signifies potentiation, <−20 antagonism, and −20 to +20 additive [29].

**Table 3:** Diagnostic doses and co-toxicity of l-carvone, l-menthone, and l-fenchone with methomyl against *Aedes aegypti*.

	1-h% Mean Knockdown ± SEM				24-h% Mean Mortality ± SEM				48-h% Mean Mortality ± SEM			
	Methomyl Alone	Synergist Alone	Mixture	Co-Toxicity Factor ^a^	Methomyl Alone	Synergist Alone	Mixture	Co-Toxicity Factor ^a^	Methomyl Alone	Synergist Alone	Mixture	Co-Toxicity Factor ^a^
2 μg applied												
Control (ethanol)	NA	0 ± 0	NA	NA	NA	0.3 ± 0.1	NA	NA	NA	0.5 ± 0.1	NA	NA
PBO	87.5 ± 9.5	7.5 ± 2.5	90 ± 10	−5.3	57.5 ± 25.3	12.5 ± 6.3	80 ± 5.8	14.3	65 ± 21.8	15 ± 3.4	96.7 ± 3.3	**20.9**
Carvone	87.5 ± 9.5	5 ± 5	96.7 ± 3.3	4.5	57.5 ± 25.3	5 ± 2.9	76.7 ± 6.7	**23**	65 ± 21.8	5 ± 2.9	86.7 ± 3.3	**24**
Menthone	87.5 ± 9.5	7.5 ± 4.8	100 ± 0	0	57.5 ± 25.3	5 ± 2.9	73 ± 3.3	**29**	65 ± 21.8	7.5 ± 2.5	80 ± 5.8	7
Fenchone	87.5 ± 9.5	0 ± 0	83.3 ± 3.3	−5	57.5 ± 25.3	2.5 ± 2.5	53.3 ± 17.6	−11	65 ± 21.8	7.5 ± 4.8	60 ± 11.5	−17
10 μg applied												
Control (ethanol)	NA	0 ± 0	NA	NA	NA	0.3 ± 0.1	NA	NA	NA	0.5 ± 0.1	NA	NA
PBO												
Carvone	87.5 ± 9.5	20 ± 5.8	100 ± 0	−7	57.5 ± 25.3	7.5 ± 2.5	83.3 ± 8.8	**28**	65 ± 21.8	15 ± 2.9	86.6 ± 8.8	8
Menthone	87.5 ± 9.5	12.5 ± 4.8	100 ± 0	0	57.5 ± 25.3	15 ± 5	93 ± 6.7	**29**	65 ± 21.8	22.5 ± 7.5	93.3 ± 6.7	7
Fenchone	87.5 ± 9.5	10 ± 4.1	93.3 ± 6.7	−4	57.5 ± 25.3	2.5 ± 2.5	73.3 ± 12	**22**	65 ± 21.8	10 ± 4.1	80 ± 10	7

^a^ A co-toxicity factor of >+20 signifies potentiation, <−20 antagonism, and −20 to +20 additive [29]. Bold numbers represent synergism according to these definitions.

**Table 4:** Oral and dermal LD_50_ values for l-menthone, l-fenchone, and l-carvone compared to permethrin and methomyl in rats and rabbits.

**Compound**	**Oral (Animal)**	**Dermal (Animal)**	**Citation**
l-menthone	500 (rt)	-	[31]
l-fenchone	6160 (rt)	5000 (rb)	[32,33]
l-carvone	5400 (rt)	>4000 (rt)	[34]
Permethrin	430–4000 (rt)	2000 (rb)	[35,36]
Methomyl	17–24 (rt)	5880 (rb)	[37]

All LD_50_ values are in mg compound per kg body weight. Animal type: rt—rat; rb—rabbit.

## Data Availability

Data are available by request through the authors.

## Sample Availability

Samples of the compounds are readily available through numerous vendors.
